# Oral Microbiome–Host Epigenetic Interactions in Oral Carcinogenesis: A Molecular Perspective

**DOI:** 10.3390/ijms27219791

**Published:** 2026-10-31

**Authors:** Ozge Unlu, Mehmet Demirci

**Affiliations:** 1 Department of Medical Microbiology, Faculty of Medicine, Istanbul Atlas University, 34408 Istanbul, Türkiye; 2 Department of Medical Microbiology, Faculty of Medicine, Kırklareli University, 39100 Kirklareli, Türkiye; mehmetdemirci@klu.edu.tr

**Keywords:** oral squamous cell carcinoma, oral microbiome, epigenetic reprogramming, tumor microenvironment, immune evasion, salivary biomarkers

## Abstract

Oral squamous cell carcinoma is still found late, and the tumor microenvironment is complicated enough that treatment often fails even when the diagnosis is timely. While genetic mutations partially account for this pathogenesis, they do not fully explain tumor behavior. Attention has therefore shifted to the microbial community around the tumor, which appears to contribute to malignancy less through the genome than through the host epigenome. This review is about the mechanism rather than about cataloging which species are present. We follow what *Porphyromonas gingivalis*, *Fusobacterium nucleatum* and *Candida albicans* do to DNA methylation, histone modification and non-coding RNA expression in host cells. While chronic inflammation plays a well-established role, bacterial and fungal metabolites also reach chromatin directly, and the resulting changes silence tumor suppressors and release oncogenic signaling in epithelium and stroma alike. We then ask what this means for tumor heterogeneity, for immune escape, and for progression, and we close with the two clinical questions that follow: whether salivary microbial and methylation signatures are usable for early detection, and whether epigenetic drugs can be aimed at the microbial component of the oral tumor microenvironment. In conclusion, the oral microbiome–epigenome axis acts as a crucial driver of tumor progression and microenvironmental immunosuppression. While translating these mechanistic insights into clinical practice necessitates rigorous in vivo validation, integrating microbiota-directed interventions with epigenetic therapies represents a highly promising frontier for the early detection and comprehensive management of OSCC.

## 1. Introduction

Oral squamous cell carcinoma (OSCC) recurs often, varies considerably from one tumor to the next, and is usually diagnosed late, which is the main reason five-year survival has remained stagnant [1]. It accounts for roughly 6% of cancer cases worldwide each year and is largely a disease of people over 50, with men affected about twice as often as women (4% versus 2%) [2,3]. Oral and pharyngeal malignancies rank tenth in cancer mortality globally. Of the 377,713 new cases recorded in 2020, close to half ended in death [4,5].

Tobacco, alcohol and high-risk human papillomavirus (HPV) infection account for a large share of that risk, and none of this is disputed. What these exposures do not account for is why patients with comparable histories follow such different molecular courses, or why some tumors respond to treatment while others do not [4,6].

For a long time OSCC was described almost entirely as a sequence of somatic genetic events: loss of tumor suppressors such as *TP53*, amplification of oncogenes, and so on. That framing has since widened. Nonmutational epigenetic reprogramming and polymorphic microbiomes both appear in the current list of cancer hallmarks as enabling characteristics [7]. DNA methylation, histone tail modification and non-coding RNA regulation change gene expression without touching the underlying sequence, and they give pre-malignant epithelium a plasticity that mutation alone cannot [7]. Within the context of this review, we strictly define ‘epigenetic mechanisms’ as these core chromatin and transcriptomic regulatory events. In contrast, classical intracellular signaling cascades (e.g., PI3K/AKT, Wnt/β-catenin, YAP) and immune evasion tactics (e.g., PD-L1 expression) are discussed herein either as the downstream phenotypic consequences of these epigenetic alterations or as the upstream inflammatory triggers that initiate chromatin remodeling.

The oral cavity runs from the vermilion border of the lips back to the posterior third of the tongue and the junction of the hard and soft palate. Because the same tissue is used for speech and swallowing, tumors here damage function early, and quality of life falls sharply even when the lesion is small [4]. This region also carries the second most complex microbial ecosystem in the body. Sequencing studies describe the tumor-associated shifts in that ecosystem as a structured, dysbiotic reorganization that tracks with disease rather than as opportunistic colonization of already damaged tissue [6], which sets up a continuous exchange between microbes and host cells.

That oral pathobionts are pro-inflammatory and can be mutagenic is well established. How a dysbiotic community is mechanistically associated with alterations in the host epigenome, step by step, is not. This review follows that axis: which organisms and which of their products reach chromatin, what they change once they arrive, and what those changes do to tumor suppressor signaling and to the surrounding microenvironment.

## 2. The Dysbiotic Shift in the Oral Tumor Microenvironment

The move from healthy mucosa through oral potentially malignant disorders (OPMD) to established OSCC is accompanied by a gradual and fairly reproducible reorganization of the resident microbiota [4,8]. Recent studies demonstrate that this reorganization is not a passive consequence of tissue damage. It appears to contribute to transformation by creating local conditions, inflammatory, mutagenic and metabolic, that favor it [9].

High-throughput sequencing of broader head and neck squamous cell carcinoma (HNSCC) cohorts consistently shows two things: commensal diversity falls, and a subset of anaerobic pathobionts is selectively enriched [9]. While these HNSCC datasets provide valuable macroscopic insights, extrapolating this evidence directly to the distinct OSCC microenvironment requires caution. Addressing this specificity, in one clinical series, tumor tissue specifically from patients with OSCC carried significantly more periodontopathogens than tissue from healthy controls [6]. Findings of this kind are what connect long-standing periodontal inflammation with oral malignancy, although they remain associations. Among the enriched organisms, *Fusobacterium nucleatum* acts as a key mediating organism; its adhesins let it co-aggregate with red-complex anaerobes, cross the epithelial barrier and set off intracellular signaling in host cells [10].

The dysbiotic community also changes local chemistry. Comparative metabolomic profiling across the OPMD-to-OSCC spectrum shows that the biochemical profile of the microenvironment shifts as dysbiosis becomes established [8]. Short-chain fatty acid ratios change, polyamines and reactive nitrogen species accumulate, and amino acid catabolites with immunomodulatory activity appear; local pH falls, oxidative stress rises, and epithelial genomic integrity suffers [8]. Some of these products act on cellular signaling directly. Others alter the substrate pools that chromatin-modifying enzymes depend on, which is where the epigenetic argument begins [8].

Once dysbiosis is treated as a contributing factor rather than a bystander, two clinical questions follow: can it be measured, and can it be modified [11]? Because particular microbial signatures track with malignant transformation, several groups have applied machine learning to salivary and tissue profiles in order to estimate oral cancer risk [12]. Deep learning applied to high-dimensional multi-omics data can pick up dysbiotic and metabolic patterns before histopathological change becomes visible [12]. Whether such models maintain predictive validity in independent populations is still open, but the underlying claim is defensible: dysbiosis is measurable, quantifiable and biologically active rather than incidental.

## 3. Pathways of Pathobiont-Associated Epigenetic Alteration

### 3.1. DNA Methylation: Microbiome-Associated Reprogramming of the Host Methylome

Multi-omics work over the past few years has moved oral dysbiosis from bystander to participant in the epigenetic instability seen in OSCC [13,14]. The host methylome in the oral tumor microenvironment is broadly redrawn, with hypermethylation concentrated at tumor suppressor promoters and hypomethylation at the regulatory regions of oncogenes [13]. Both directions appear together, which is what makes the pattern look programmed rather than random [13,14]. Bacterial infection can disturb methylation in host cells directly, and because these changes often precede the major mutational events, they sit early in the sequence [14]. Spatial epigenomic methods now allow methylation to be mapped in situ across tumor tissue. Those maps show that aberrant promoter methylation is patchy, shaped both by the microbiota and by crosstalk with cancer-associated fibroblasts (CAFs), and that the resulting pattern relates to perineural invasion (PNI) and to worse prognosis [15].

A short list of keystone pathobionts accounts for most of the reported correlations with host methylation change: *Porphyromonas gingivalis*, *Fusobacterium nucleatum*, *Peptostreptococcus simiae*, *Catonella morbi* and *Treponema medium* [13,14]. The reported effects extend beyond local inflammation, since these species are associated with methylation changes that silence tumor suppressors and release oncogenic signaling [13,14].

#### 3.1.1. Hypermethylation of Tumor Suppressors

Bacterial load within the tumor niche correlates with CpG methylation at specific promoter regions [13]. For example, in human OSCC cohorts, an increased abundance of *C. morbi* correlates with dense hypermethylation and transcriptional silencing of PITX1 and AOX1 [13]. Losing PITX1 removes a regulatory brake on the RAS pathway; loss of AOX1 expression has been associated with invasion and metastasis [13]. *P. gingivalis* has a comparable effect on CDKN2A (p16) and E-cadherin, suppressing both and loosening cell cycle control [14]. Its lipopolysaccharide (LPS) also reduces MLH1 expression through promoter hypermethylation, which impairs mismatch repair (MMR) and produces something resembling a microsatellite instability phenotype [14]. Interleukin-6 (IL-6) appears to be the intermediate step: *P. gingivalis* LPS drives a sharp rise in IL-6, and IL-6 in turn drives both the promoter hypermethylation and the accompanying genome-wide hypomethylation. Because hypermethylation of CDKN2A, hMLH1 and hMSH2 is among the earliest detectable events in OSCC, these marks are now being tested as non-invasive salivary biomarkers for early detection and risk stratification [16]. *F. nucleatum* and *P. simiae*, both structural members of the biofilm, are likewise associated with hypermethylated loci across the OSCC genome [13].

#### 3.1.2. Hypomethylation and Oncogene Activation

The reverse also occurs. Similarly, enrichment of T. medium correlates inversely with CpG methylation at the NRG1 promoter, which is associated with concurrent NRG1 overexpression [13]. NRG1 binds and activates ErbB receptor tyrosine kinases, feeding into PI3K/AKT and MAPK/ERK signaling [13]. ITGB4, an integrin implicated in adhesion loss and in aggressive squamous disease, is upregulated the same way, in that case alongside *C. morbi* enrichment [13]. Similar stromal responses have been observed in periodontitis models. Gingival fibroblasts exposed to *P. gingivalis* in vitro undergo DNA hypomethylation, and their output of intercellular adhesion molecule-1 (ICAM-1) and of MMP1, MMP9 and MMP13 rises. Matrix breaks down, invasion becomes easier, and because ICAM-1 supports bacterial adhesion, more bacteria attach: a feed-forward loop [17]. Taken together, these targeted methylome changes offer a mechanism for how particular oral pathobionts shift host gene expression toward a tumor-permissive state [13,14]. They do not act alone. Systemic immune changes run in parallel, with species such as *Staphylococcus aureus* and *Gemella haemolysans* associated with raised IL-22 and CCL5, which has been proposed as the systemic link that keeps the local niche inflamed [18].

### 3.2. Histone Modification and Chromatin Remodeling: Microbial Metabolites and Structural Hijacking

Chromatin structure is the second point of contact. Acetylation, methylation and phosphorylation of histone tails determine how accessible DNA is to transcription, and in OSCC these marks are redistributed in ways that favor inflammatory mediators and proto-oncogenes [16].

#### 3.2.1. Bacterial Metabolites as Endogenous HDAC Inhibitors

Short-chain fatty acids (SCFAs), butyrate in particular, inhibit histone deacetylases (HDACs). HDACs normally strip acetyl groups from histones and keep chromatin compact; when butyrate accumulates in the tumor microenvironment, that baseline regulation is lost [14]. The situation in OSCC is complicated by the fact that HDAC1 and HDAC2 are themselves overexpressed in many tumors, where they repress p53 and stabilize HIF-1α [19]. Bacterial SCFAs compete with this activity and push chromatin toward an open, hyperacetylated state [14]. Transcription of genes involved in chronic inflammation and cell cycle progression increases accordingly. The effect reaches the HDAC–NF-κB axis and, through it, epithelial–mesenchymal transition (EMT), where altered E-cadherin expression determines how motile and invasive the cells become [20]. There is a further parallel with pharmacological HDAC inhibition: enhancer and super-enhancer landscapes are deregulated, transcriptomic heterogeneity is sustained, and master regulators such as c-Myc are aberrantly activated, which allows tumor subclones to adapt to local stress [21]. Lactate adds another layer. Produced both by dysbiotic microbial metabolism and by hypoxic tumor cells, it is the precursor for histone lactylation at sites such as H3K18la and H4K8la. Lactylation holds chromatin open, supports β-catenin-dependent transcription and pushes macrophages toward an immunosuppressive, pro-tumorigenic phenotype [22]. HDAC6 contributes to the same immunosuppressive picture from the host side, inducing IL-13 through the AP-1 pathway, driving M2 polarization and reducing sensitivity to cisplatin [19]. It is crucial to note that the role of butyrate is highly context-dependent, a phenomenon often described as the ‘butyrate paradox.’ In healthy, non-transformed epithelium, low concentrations of butyrate are rapidly metabolized in the mitochondria via beta-oxidation, serving as a primary energy source. However, in malignant cells that undergo metabolic reprogramming toward aerobic glycolysis (the Warburg effect), butyrate is not efficiently metabolized. Consequently, it accumulates in the nucleus, where it reaches concentrations sufficient to inhibit specific HDAC isoforms (primarily Class I and IIa). Furthermore, the impact of butyrate exhibits cell-type specificity; while it may promote pro-tumorigenic open chromatin states in genetically damaged epithelial cells, it can simultaneously exert anti-inflammatory effects on local immune cells. Importantly, much of the mechanistic evidence regarding this paradox is extrapolated from colorectal cancer and periodontitis models. Direct in vivo demonstration of these specific SCFA dynamics within the OSCC microenvironment remains an area requiring further investigation [19,21].

#### 3.2.2. Targeted Histone Phosphorylation by Keystone Pathobionts

Some pathobionts act on histones more directly. Both *F. nucleatum* and *P. gingivalis* induce phosphorylation of histone H2A in oral epithelial cells [14,16]. The modification alters chromatin in a way that suppresses p53 and Ku70 signaling, so DNA repair and apoptosis are weakened at once [16]. Cells that have lost those two safeguards survive damage they should not survive, and they tolerate treatment better than they should. This structural change also overlaps with the metabolic route described above, since HDAC inhibition hyperacetylates p53 and Ku70 as proteins rather than only as transcriptional targets. The two effects converge on the same repair pathways, non-homologous end joining and homologous recombination, and genomic instability increases [21]. Spatial multi-omics adds an important qualification: these microbially driven histone changes are unevenly distributed within a tumor. That unevenness contributes to intratumoral heterogeneity and is associated with perineural invasion and poorer outcomes [15].

### 3.3. Non-Coding RNA Dysregulation: Microbiome-Host Crosstalk After Transcription

Non-coding RNAs are the third layer. MicroRNAs (miRNAs) and long non-coding RNAs (lncRNAs) set the post-transcriptional balance between proliferation and apoptosis and shape immune signaling, and dysbiotic communities interfere with them as well, producing what has been described as an “epigenetic modification–ncRNA–target gene” cascade [14,22].

#### 3.3.1. Pathobiont-Specific Changes in miRNA Profiles

Preclinical models indicate that *F. nucleatum* and *P. gingivalis* are associated with increased expression of miR-21 and miR-31 [16,23]. miR-21 is the better characterized of the two. Working through the miR-21/PDCD4/AP-1 axis, it silences Programmed Cell Death 4 (PDCD4) and PTEN post-transcriptionally, and the result is resistance to apoptosis alongside unchecked epithelial proliferation [16,23].

Protective miRNAs are suppressed in parallel. Exposure to *P. gingivalis* lowers miR-125a and miR-200a, both already reduced in OSCC patients and both important for epithelial homeostasis [16]. The wider periodontitis-associated consortium shifts a larger set: miR-155, miR-224 and miR-210 up, miR-497 and miR-29c down [14,16]. miR-155 and miR-146a are particularly notable because they feed back into NF-κB, which sustains the inflammation the whole process depends on. Suppression of the miR-200 family together with loss of miR-34 pushes cells toward EMT and greater invasive capacity [24,25]. Clinically, raised miR-21 and miR-155 track with tumor growth and also with acquired chemotherapy resistance, which is what makes them candidate therapeutic targets [24]. Transcriptomic surveys place these observations on familiar ground: individual miRNAs act as oncomiRs or as suppressors depending on context, and most converge on p53 and PI3K/AKT signaling [25]. Competing endogenous RNA (ceRNA) networks complicate the picture further, since transcripts sharing microRNA response elements titrate one another’s availability [26].

#### 3.3.2. Metabolite-Driven lncRNA Dysregulation

Microbial metabolites reach this network too. SCFA-mediated HDAC inhibition changes histone acetylation at lncRNA promoters [22], and one consequence is altered expression of HOTAIR, a well-documented driver of EMT and stem-cell-like behavior in squamous malignancies [22]. Downstream, several of these lncRNAs, H19 and MALAT1 among them, work as molecular sponges, sequestering tumor-suppressive miRNAs and freeing the oncogenic transcripts those miRNAs would otherwise hold down [25]. Local metabolic state is therefore tied to chromatin accessibility at ncRNA promoters, and this regulatory feedback continuously reinforces the malignant phenotype [25].

## 4. Mechanistic Contributions of Specific Pathobionts

The shift from a stable oral microbiota to a pro-inflammatory community involves interactions between species, not simply the arrival of new ones [27]. Chronic periodontitis is the clearest model: sustained host-pathobiont interaction and failure to resolve inflammation destroy tissue and change the local niche [28,29]. In carcinogenesis, several pathobionts enriched in malignant tissue alter epithelial physiology through their own virulence factors and metabolic products, and those interactions produce specific epigenetic and signaling changes in the host [6].

### 4.1. Porphyromonas gingivalis: Epigenetic Reprogramming, Immunoevasion and Prognosis

*Porphyromonas gingivalis* is an obligate anaerobic Gram-negative organism and the keystone pathobiont of severe periodontitis. It is consistently enriched within the OSCC tumor microenvironment [30], and it contributes to oncogenesis along several routes at once: remodeling of the tumor microenvironment (TME), interference with host epigenetic machinery, and suppression of local anti-tumor immunity [31].

Attachment to epithelium uses fimbriae (FimA). Once inside the cell, *P. gingivalis* avoids autophagic destruction and activates PI3K/Akt to block apoptosis, so epithelial cells carrying genetic damage stay alive [32,33]. Its metabolic byproducts, again mainly butyrate, act as endogenous HDAC inhibitors [32]. Chromatin opens, transcription of cell cycle genes changes, and with prolonged infection DNA methyltransferase (DNMT) dynamics shift as well, producing promoter hypermethylation and silencing of tumor suppressor genes including *CDKN2A* [30].

Immune evasion runs alongside this. *P. gingivalis* shields oral cancer cells from macrophage-mediated cytotoxicity [33] and interferes with antigen presentation by upregulating Programmed Death-Ligand 1 (PD-L1) on dendritic cells, which paralyzes CD8+ T-cell effector function [34]. Co-aggregation with *F. nucleatum* amplifies the effect and maintains the pro-tumorigenic cytokine profile of the microenvironment [35].

**Figure 1:**
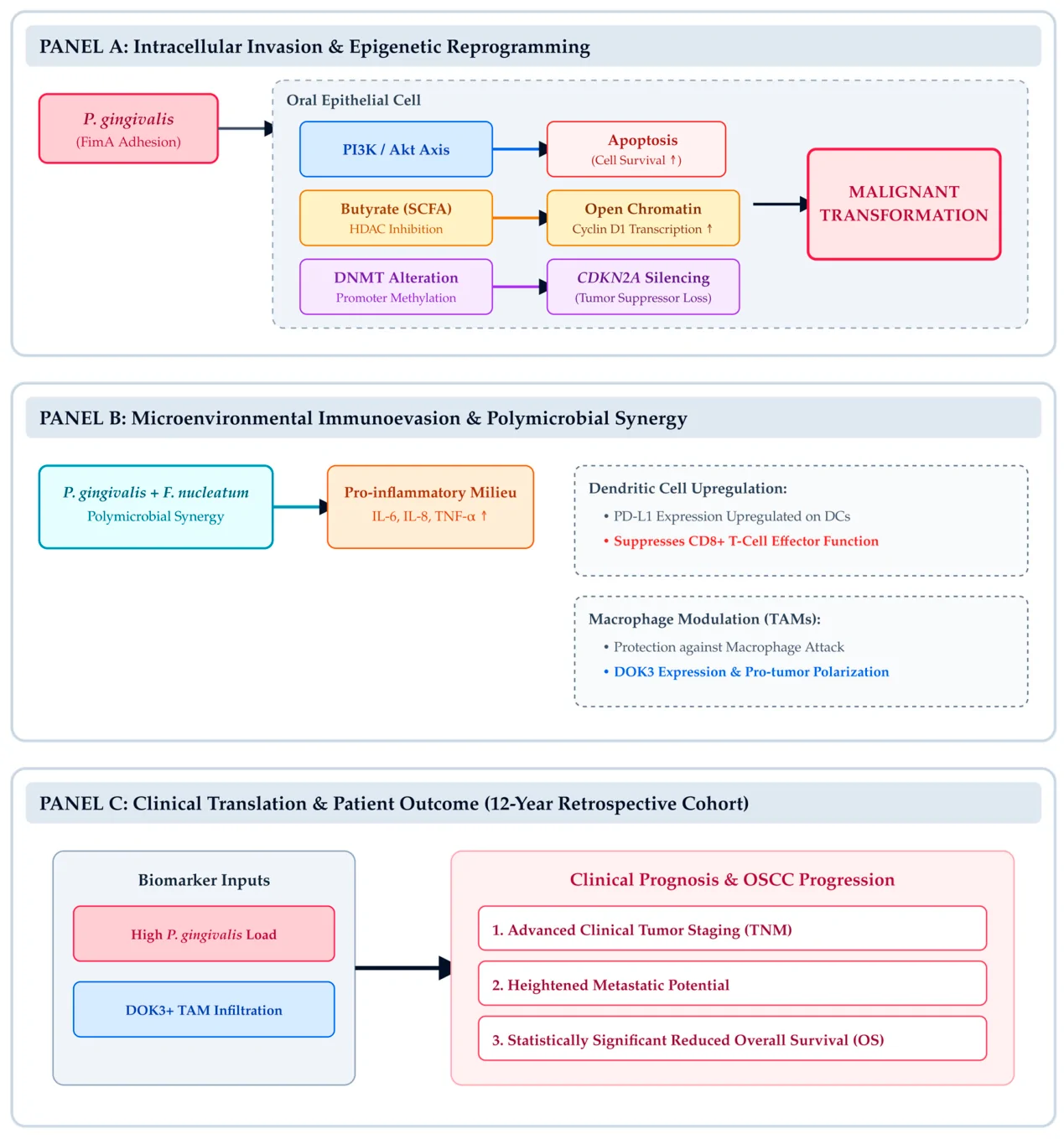
*Porphyromonas gingivalis*-driven oral carcinogenesis. (**A**) Intracellular epigenetic reprogramming: *P. gingivalis* uses *FimA* fimbriae to adhere to and invade oral epithelial cells, then activates anti-apoptotic PI3K/Akt signaling to keep the host cell alive. Bacterial short-chain fatty acids, mainly butyrate, inhibit HDACs and force chromatin open. The resulting change in DNA methyltransferase (DNMT) kinetics produces promoter hypermethylation that silences tumor suppressor genes, including CDKN2A. (**B**) Microenvironmental immunoevasion: within the tumor microenvironment (TME), *P. gingivalis* co-aggregates with *Fusobacterium nucleatum* and sustains a chronic pro-tumorigenic cytokine cascade. By inducing PD-L1 expression on dendritic cells it blunts cytotoxic CD8+ T-cell responses, and it polarizes tumor-associated macrophages (TAMs) toward an immunosuppressive phenotype, shielding malignant cells from immune clearance. (**C**) Clinical prognosis: high intratumoral *P. gingivalis* load, together with particular TAM infiltration profiles and DOK3 expression, correlates with advanced staging, metastasis, and poor overall survival. Note: Solid arrows in this diagram denote proposed directional associations, extrapolations from periodontitis models, and mechanistic hypotheses derived from the current literature, rather than definitively proven linear causal pathways in human OSCC.

There is prognostic evidence to match the mechanism. A 12-year retrospective analysis of 200 OSCC patients found a significant association between *P. gingivalis* infection, tumor-associated macrophage (TAM) infiltration and expression of DOK3 (Docking protein 3) [36]. High bacterial load combined with particular macrophage polarization profiles correlated with advanced staging, metastasis and poor overall survival [36]. Detecting the organism and its associated immune signature may therefore carry prognostic value, and may point to targets in patients whose tumors resist immunotherapy. As a theoretical framework derived from these associative and preclinical findings, Figure 1 schematizes the proposed mechanisms of *Porphyromonas gingivalis*-driven oral carcinogenesis.

### 4.2. Fusobacterium nucleatum: Adhesin-Mediated Signaling and Non-Coding RNA Dysregulation

*Fusobacterium nucleatum* is an anaerobic Gram-negative pathobiont and the main bridging organism in subgingival biofilm. In OSCC it has a functional role in initiation, progression and metastatic dissemination [37,38]. In broader HNSCC cohorts, higher tissue abundance goes with lymph node involvement, advanced clinical staging and shorter overall survival. Although comprehensive OSCC-specific prognostic data is still maturing, these trends strongly suggest its pathogenic role. Rather than colonizing neoplastic tissue opportunistically, it uses specialized outer membrane proteins to engage host receptors, trigger intracellular signaling and change gene expression in epithelial and immune cells alike [23].

*Fusobacterium* adhesin A (FadA) is the primary virulence factor at the epithelial surface. It binds the extracellular domain of E-cadherin, dismantles intercellular junctions and allows rapid bacterial internalization [39]. β-catenin is phosphorylated and translocates to the nucleus, Wnt/β-catenin signaling stays constitutively active, and downstream targets including matrix metalloproteinases (MMPs), MYC and CCND1 (Cyclin D1) are upregulated [37].

A second route runs through YAP. Intracellular *F. nucleatum* promotes the dephosphorylation and nuclear accumulation of Yes-associated protein, the principal transcriptional co-activator of the Hippo pathway, which supports epithelial stemness, proliferation and resistance to apoptosis [40]. While these signaling cascades (Wnt/β-catenin and YAP) represent downstream functional consequences of microbial invasion, *F. nucleatum* also directly manipulates core epigenetic machinery by raising host miR-21 and miR-31, silencing PTEN and PDCD4 post-transcriptionally [23].

Fap2, the secondary adhesin, is what makes the microenvironment immunosuppressive. It binds TIGIT (T-cell immunoreceptor with Ig and ITIM domains) on natural killer (NK) cells and tumor-infiltrating lymphocytes (TILs), and that engagement suppresses cytotoxic killing of tumor cells [23,39]. Persistent infection also drives EMT through the familiar cadherin switch, N-cadherin up and E-cadherin down, with the expected effects on motility, invasion and distant spread [23,39]. As for treatment response, intratumoral *F. nucleatum* induces autophagy-mediated resistance to 5-fluorouracil and cisplatin by activating Toll-like receptor 4 (TLR4) and modulating specific microRNA networks [23].

**Figure 2:**
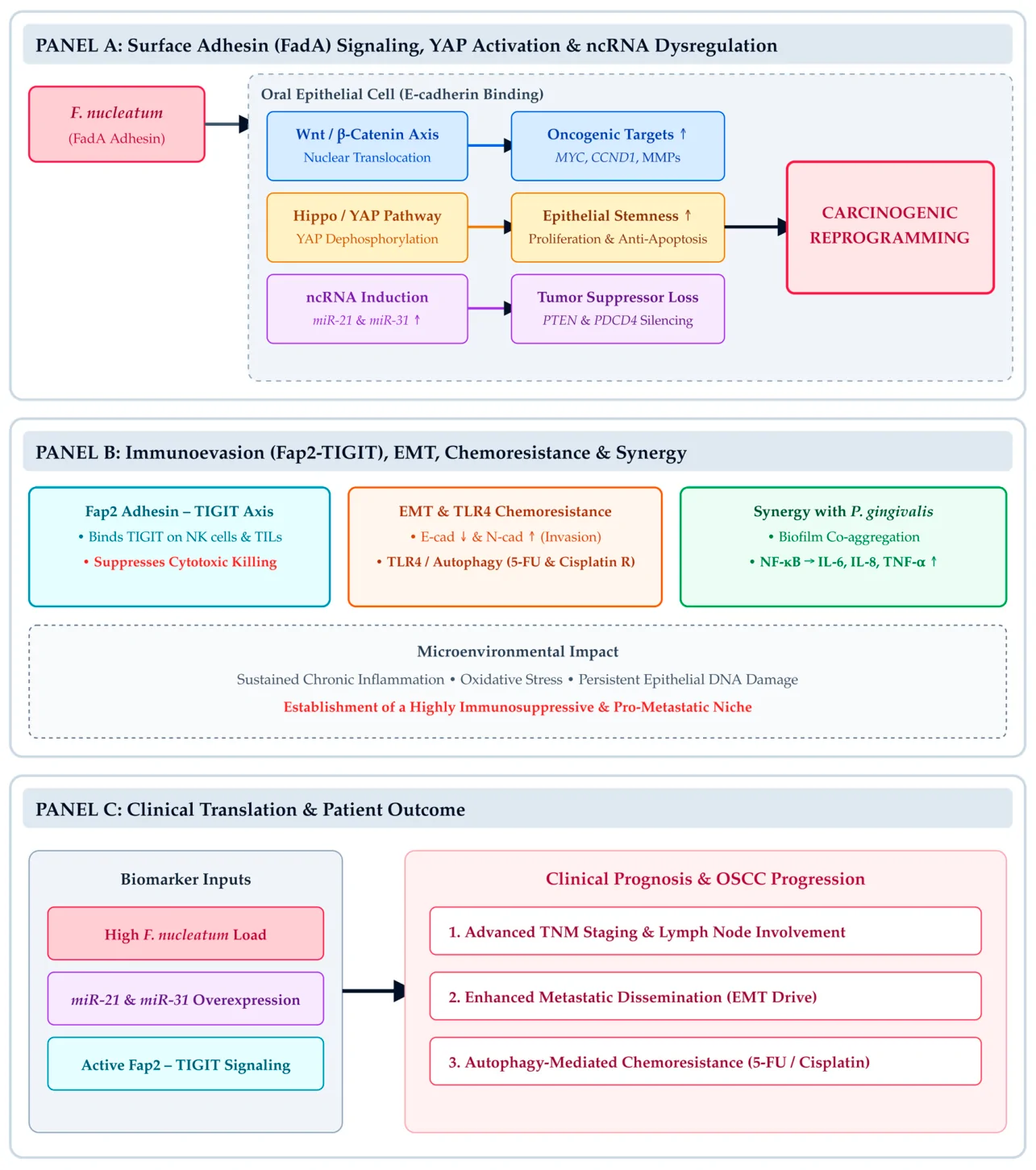
*Fusobacterium nucleatum*-mediated signaling and oncogenesis. (**A**) Adhesin-mediated epithelial signaling, YAP activation and ncRNA dysregulation: the outer membrane adhesin FadA binds the extracellular domain of E-cadherin on oral epithelial cells, triggering internalization and junctional disruption. β-catenin translocates to the nucleus, Wnt/β-catenin signaling becomes constitutive, and oncogenic targets including *MYC*, *CCND1* (Cyclin D1) and matrix metalloproteinases (MMPs) are upregulated. Intracellular *F. nucleatum* also promotes dephosphorylation and nuclear accumulation of Yes-associated protein (YAP), supporting epithelial stemness, proliferation and anti-apoptotic survival, and it upregulates host non-coding RNAs (*miR-21*, *miR-31*) that silence *PTEN* and *PDCD4*. (**B**) Immunoevasion, EMT, TLR4-driven chemoresistance and polymicrobial synergy: the secondary adhesin Fap2 binds TIGIT on natural killer (NK) cells and tumor-infiltrating lymphocytes (TILs), suppressing cytotoxic tumor lysis. Persistent infection drives epithelial-to-mesenchymal transition (EMT) through a cadherin switch, with E-cadherin suppressed and N-cadherin upregulated. Through TLR4 activation and microRNA modulation, *F. nucleatum* promotes autophagy-mediated resistance to 5-fluorouracil (5-FU) and cisplatin. Co-aggregation with *P. gingivalis* activates host NF-κB signaling, and the resulting IL-6, IL-8 and TNF-α establish a persistent oxidative microenvironment. (**C**) Clinical prognosis: high intratumoral *F. nucleatum* density, elevated *miR-21*/*miR-31* expression and active Fap2-TIGIT engagement correlate with advanced TNM staging, lymph node metastasis, chemoresistance and reduced overall survival (OS) in OSCC patients. Note: Solid arrows indicate proposed mechanistic interactions and functional associations based on in vitro and associative clinical data, and do not imply definitive standalone causality in OSCC.

Its oncogenic capacity increases substantially when *P. gingivalis* is present [35,38]. Surface lectin–carbohydrate interactions allow the two organisms to co-aggregate, which improves mucosal retention, tissue invasion and biofilm stability for both [35]. Co-infection then sustains a hyper-inflammatory state, with elevated interleukin-6 (IL-6), interleukin-8 (IL-8) and tumor necrosis factor-alpha (TNF-α) secreted via NF-κB activation [35]. The accompanying oxidative stress and metabolic change favor DNA damage and epigenomic alteration in the adjacent epithelium [35,38]. Reflecting these proposed interactions rather than standalone clinical causality, Figure 2 demonstrates the hypothesized *Fusobacterium nucleatum*-mediated signaling and oncogenesis.

### 4.3. Candida albicans: Metabolic Carcinogenesis, Fungal–Bacterial Interaction and Immune Modulation

Bacteria dominate periodontal dysbiosis, but *Candida albicans* is a frequent member of the polymicrobial biofilms found at malignant lesions [4]. Its pathogenicity rests on the dimorphic switch from yeast to invasive hyphal forms and on candidalysin, a peptide toxin that has been shown in in vitro oral epithelial models to compromise epithelial membranes and trigger epidermal growth factor receptor (EGFR) signaling [41].

Its metabolic contribution is more specific. *C. albicans* expresses alcohol dehydrogenases that convert ethanol into acetaldehyde, a mutagen capable of forming DNA adducts and disrupting repair machinery [41]. In in vitro mixed-species biofilm models containing *P. gingivalis* and *F. nucleatum*, the fungus stabilizes biofilm architecture, and the three organisms together sustain the release of reactive oxygen species (ROS) [4,42]. Drawing inferences from broader epigenetic studies in other tissues, long-term ROS exposure is known to alter the kinetics of DNMTs and Ten-Eleven Translocation (TET) enzymes. While this suggests a potential mechanism for shifting methylation locally in oral epithelial stem cell populations, this exact epigenetic sequence remains a theoretical mechanism that has yet to be directly validated in vivo within the OSCC microenvironment.

**Figure 3:**
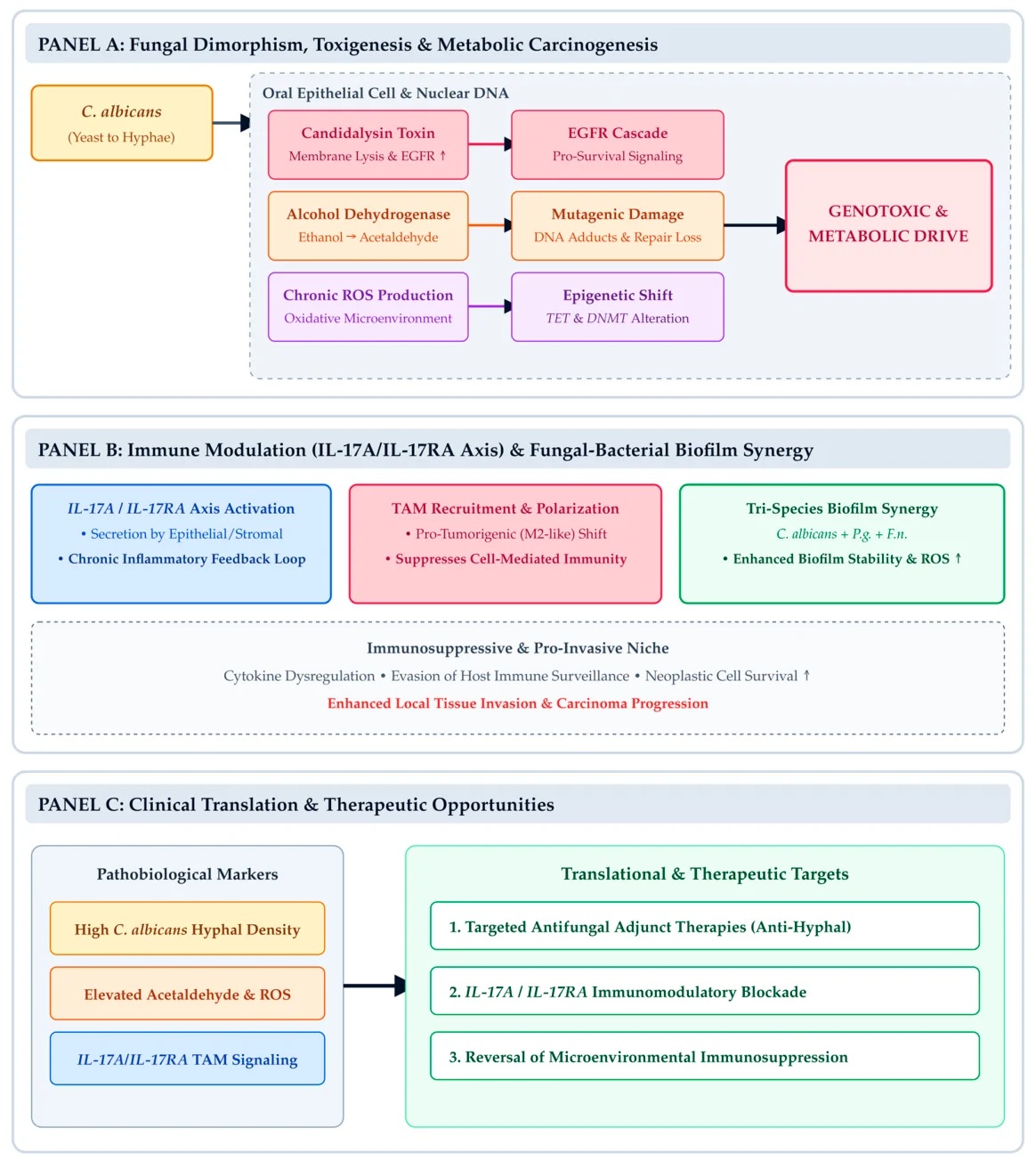
*Candida albicans*-driven metabolic carcinogenesis and immune modulation. (**A**) Fungal dimorphism, candidalysin toxigenesis and metabolic carcinogenesis: the transition from yeast to invasive hyphal forms allows cellular penetration and release of candidalysin, a peptide toxin that destabilizes epithelial membranes and triggers EGFR pro-survival signaling. Fungal alcohol dehydrogenases convert ethanol into acetaldehyde, a mutagen that forms DNA adducts and damages host repair systems. Chronic reactive oxygen species (ROS) accumulation impairs Ten-Eleven Translocation (TET) and DNA methyltransferase (DNMT) catalytic kinetics, driving aberrant methylation within epithelial stem cell niches. (Panel (**B**)) Immune modulation and polymicrobial synergy: *C. albicans* activates the host *IL-17A/IL-17RA* axis in the oral mucosa. Local *IL-17A* binding to *IL-17RA* on epithelial and stromal cells recruits tumor-associated macrophages (TAMs) and polarizes them toward a pro-tumorigenic phenotype, creating a chronic inflammatory feedback loop. Within mixed biofilms, *C. albicans* acts synergistically with *Porphyromonas gingivalis* and *Fusobacterium nucleatum*, stabilizing biofilm architecture, amplifying ROS generation, suppressing cytotoxic immunity and enhancing neoplastic invasion. (Panel (**C**)) Translational and therapeutic opportunities: high intratumoral hyphal density, elevated acetaldehyde levels and active *IL-17A/IL-17RA* signaling mark therapeutic vulnerabilities that could be addressed through antifungal adjuncts targeting hyphal transition and through selective *IL-17A/IL-17RA* immunomodulation. Note: The pathways depicted represent hypothesized mechanistic sequences and microenvironmental associations deduced from mixed-biofilm and mucosal models. Arrows indicate proposed directional flow rather than established linear causation in OSCC.

There is an immunological arm as well. Evidence from mucosal infection models indicates that *C. albicans* activates the host IL-17A/IL-17RA axis in the oral mucosa: fungal colonization induces local interleukin-17A, which binds IL-17RA on epithelial and stromal cells and recruits TAMs, polarizing them toward a pro-tumorigenic phenotype [43]. Sustained signaling through this axis maintains an inflammatory feedback loop, suppresses cell-mediated immunity, and increases both the invasive capacity and the survival of malignant cells in these models [43,44]. Consequently, the potential use of targeted antifungal adjuncts and IL-17-directed immunomodulation represents an intriguing preclinical hypothesis for future investigation rather than a near-term therapeutic strategy. Rigorous clinical studies will be necessary to determine if manipulating this fungal-immune axis can effectively restore microenvironmental homeostasis in OSCC patients [44]. The proposed theoretical pathways for *Candida albicans*-driven metabolic carcinogenesis and immune modulation are outlined in Figure 3.

## 5. Immune Evasion and Tumor Progression Along the Epigenome-Microbiome Axis

The oral TME is an immunological compartment as much as a site of tissue destruction, and the dysbiotic microbiome and host epigenome work on it together. Structural virulence factors and metabolic byproducts reprogram the local immune compartment, and neoplastic cells become correspondingly harder for the immune system to see [13,14]. The profound immunosuppressive TME is largely a downstream consequence of the aforementioned microbiome–epigenome interactions. While physical immune evasion (e.g., PD-L1 and TIGIT engagement) operates at the cellular surface, these immunomodulatory phenotypes are deeply reinforced by underlying epigenetic reprogramming [13,14].

### 5.1. Immune Checkpoint Upregulation and T-Cell Suppression

Two mechanisms dominate the literature. Fap2 on *F. nucleatum* binds TIGIT on NK cells and TILs, delivering an inhibitory signal that suppresses cytotoxic lysis of cancer cells [14]. *P. gingivalis* works on the antigen-presenting side, raising PD-L1 expression on dendritic cells and epithelial populations, which inhibits CD8+ T-cell effector function and accelerates progression [14].

### 5.2. Macrophage Polarization and Stromal Remodeling

Innate immunity is skewed in the same direction. *P. gingivalis* infection recruits TAMs and polarizes them toward an anti-inflammatory, pro-angiogenic, and immunosuppressive phenotype. While traditionally simplified as ‘M2-like,’ modern macrophage biology recognizes that these TAMs do not adhere to a strict binary. Instead, they exhibit substantial plasticity and heterogeneity, adopting mixed phenotypes dictated by dynamic microenvironmental and microbial cues [14]. Two molecular inputs are involved: innate sensing through Toll-like receptors, and the CSF-1R axis. Outer membrane vesicles (OMVs) and pathogen-associated molecular patterns (PAMPs) released by oral pathobionts keep NF-κB and STAT3 active, raise inflammatory cytokines, drive this pro-tumorigenic M2 polarization with CD206 and CD163 on the cell surface, and elevate IL-10, TGF-β and PD-L1 [45,46]. Evidence from periodontitis models suggests that the stroma may reinforce this loop: hypomethylation and remodeling in gingival and periodontal fibroblasts increase local release of chemokines and matrix-remodeling enzymes that recruit regulatory immune cells [17]. Metagenomic work also implicates less familiar organisms, with *Staphylococcus aureus* and *Gemella haemolysans* linked to local and systemic upregulation of IL-22 and CCL5 [18].

### 5.3. Epigenetic Reprogramming and Trained Immunity

Some of this reaches beyond the local microenvironment, though evidence stems primarily from models of chronic periodontitis rather than OSCC directly. In these periodontal models, chronic exposure to oral pathobionts is associated with lasting epigenetic reprogramming in hematopoietic stem and progenitor cells (HSPCs) in bone marrow, a maladaptive form of trained immunity (TRIM). Stable chromatin remodeling and metabolic rewiring give these precursors an imprinted pro-inflammatory memory. It is hypothesized that their hyper-responsive, myeloid-biased progeny could continue to arrive at the tumor site, potentially amplifying immunosuppression [47]. Work on the so-called oral-X axis describes the same phenomenon at the level of individual marks: sustained low-grade periodontal inflammation and endotoxemia are associated with long-term changes in H3K4me3 and H3K9me3 and in chromatin accessibility at hematopoietic and monocytic gene promoters, producing a self-sustaining systemic inflammatory state [48]. We explicitly frame this ‘oral-X axis’ as an extrapolated systemic mechanism; whether these specific bone marrow-level epigenetic alterations actively fuel OSCC progression remains a compelling but unproven hypothesis.

### 5.4. Microbiome-Mediated Immunotherapy Resistance

A critical translational question is how this altered microenvironment impacts clinical treatment. Rather than definitively proving causation, the current literature supports a stratified perspective. First, there is clear evidence of microbiome-mediated immune suppression in vitro, as seen through Fap2-TIGIT engagement and PD-L1 upregulation. Second, these dysbiotic profiles show strong associations with poor prognosis in HNSCC cohorts. Third, regarding altered response to immune checkpoint inhibitors (ICIs), drawing strong conceptual parallels from established microbiome-immunoresistance models in broader oncology [20], emerging correlative studies suggest that severe oral dysbiosis may also negatively impact immunotherapy efficacy [49]. However, direct clinical evidence proving that the oral microbiome independently causes ICI resistance in OSCC patients remains an active area of investigation. Pathobiont-driven changes in the TME impair the priming and infiltration of effector CD8+ cytotoxic T cells while leaving suppressive regulatory networks intact, and the tumor ends up immunologically “cold”, which favors recurrence [49]. Multi-cohort metagenomic profiling suggests the community should be assessed as a whole rather than organism by organism; microbial risk scores (MRS) built on that principle represent an early clinical framework to stratify patients by risk of head and neck cancer and by likely response to immunotherapy [50]. If those tools hold up, microbiome modulation should be integrated with epigenetic and immunotherapeutic strategies rather than being handled separately [51].

## 6. Clinical Translation: Salivary Biomarkers and Epidrugs

Non-invasive detection is where most of the translational interest sits. Saliva reflects local oral pathology and, to some extent, systemic pathobiological processes [46], so profiling both its microbial and its epigenetic content offers significant clinical utility for a cancer that is usually diagnosed at an advanced stage [6].

### 6.1. Salivary Microbial and Epigenetic Signatures in Early Diagnosis

Saliva contains host cells, immune mediators, systemic analytes and the oral microbiome itself. Metagenomic and epigenomic investigations have produced two categories of signature.

The first is microbial. Enrichment of periodontal pathobionts such as *Porphyromonas gingivalis* and *Fusobacterium nucleatum*, or depletion of protective commensals, gives a reasonably consistent dysbiosis signature [50]. Because these organisms generate genotoxic metabolites and drive both local and systemic inflammation, their salivary detection serves as a robust early clinical paraneoplastic screening candidate [52]. The second is epigenetic. Promoter hypermethylation silencing essential tumor suppressors is a shared feature of oral and systemic carcinogenesis, and cell-free tumor DNA (cfDNA) together with modified host and microbial nucleic acids shed into saliva provides a stable, disease-specific target [25]. Methylation profiling of liquid biopsies could in principle identify malignant transformation before it becomes clinically apparent [26].

While combining host epigenetic and salivary microbial markers in a multi-parametric panel is a logical progression, this approach requires rigorous critical scrutiny. Salivary diagnostics in OSCC face significant confounding variables. A major limitation is disease specificity; a dysbiotic microbial signature detected in saliva may merely reflect concurrent periodontal inflammation or poor oral hygiene rather than true tumor biology. Furthermore, variations in smoking and alcohol consumption, recent antibiotic or medication use, age, dietary habits, and the specific tumor site within the oral cavity profoundly influence both the microbiome and the epigenome. Without strictly controlling for these confounders, salivary panels risk high false-positive rates. Therefore, prospective validation must go beyond merely calculating sensitivity; it must definitively separate the molecular background noise of chronic periodontitis and lifestyle factors from the bona fide oncogenic signals of OSCC [53].

### 6.2. Epigenetic Drugs and Microbiota-Directed Interventions

Therapeutic strategies are similarly evolving, shifting from relying solely on cytotoxicity toward interventions that remodel the microenvironment and restore epigenetic and microbial balance.

Epidrugs come first. DNA methyltransferase inhibitors (DNMTis) and histone deacetylase inhibitors (HDACis) reprogram the abnormal epigenetic state of both neoplastic and stromal compartments [51]. Reactivating silenced tumor suppressor genes was the original rationale, but the immunological effects may matter more: epigenetic drugs can reverse macrophage polarization and partly restore exhausted cytotoxic T cells, which is the basis for combining them with ICIs [49]. Clinical evidence is still in the early clinical stage. Phase II work with decitabine plus chemotherapy, and with vorinostat in chemoresistant disease, suggests the approach is viable in OSCC without yet establishing benefit [54].

Effective targeted delivery presents an additional clinical challenge. Polymeric and inorganic gold nanoparticle systems have been developed to carry epigenetic modifiers, RNA interference (RNAi) constructs and chemotherapeutic payloads with microenvironment-responsive release, largely in order to limit systemic toxicity [51].

Probiotics and postbiotics approach the same axis from the microbial side. Targeted administration of probiotics and postbiotics, including microbial SCFAs such as butyrate, has been proposed as a preclinical strategy to reverse pathobiont-induced epigenetic changes, tighten epithelial barriers and reduce low-grade systemic inflammation [45,47]. The pharmacological application of SCFA-based postbiotics presents a conceptual challenge due to the ‘butyrate paradox.’ Their therapeutic potential likely relies on concentration gradients and timing. While high local concentrations of microbially derived butyrate in established tumors might drive aberrant chromatin opening, systemic or low-dose administration in early or pre-malignant stages is hypothesized to restore epithelial barrier integrity and promote the expansion of regulatory T cells (Tregs). Therefore, careful consideration of local versus systemic exposure, as well as the metabolic state of the target tissue, is required before positioning postbiotics as viable interventions in OSCC [45,47].

Dietary phytochemicals belong in the same category. Curcumin, epigallocatechin-3-gallate, sulforaphane and berberine inhibit DNMTs and HDACs, regulate oncogenic non-coding RNAs and can re-sensitize resistant cells to conventional chemotherapy in preclinical systems. Whether any of these compounds retain efficacy and bioavailability in human clinical trials remains a standing question, and answering it requires multi-omics profiling, pharmacodynamic validation in tumor tissue and biomarker-guided trial design [55].

Salivary biosensing widens the window in which oral cancer can be detected; epidrugs combined with microbiome-directed treatment offer a way to act inside that window. Neither is ready for routine clinical use.

## 7. Conclusions and Future Directions

OSCC and the related upper aerodigestive malignancies remain difficult to treat, and the reasons are not confined to classical genomic alteration and environmental insult. The evidence reviewed here places the oral microbiome among the active contributors, working mainly through the host epigenome. Keystone pathobionts carry adhesins that interfere with immune recognition and produce metabolites that reach chromatin directly.

Through effects on host DNMT activity, metabolic inhibition of HDACs and interference with non-coding RNA networks, these organisms alter chromatin architecture, silence principal tumor suppressor pathways and help build an immunosuppressive tumor microenvironment. Some of the consequences are systemic: local periodontal dysbiosis leaves an epigenetic and inflammatory imprint that persists beyond the oral cavity and has been linked to therapeutic refractoriness and to resistance against immune checkpoint inhibitors.

### What the Field Needs Next

Four primary milestones must be achieved to translate these observations into clinical practice. The first is causality. Most of the microbiome data are correlative, and the field needs disease-specific pharmacodynamic validation, with paired tissue biopsies, liquid biopsies and spatial multi-omics confirming that the predicted epigenetic changes occur where and when the model says they should. The second is the salivary panel itself: assays combining dysbiosis signatures, host methylation and machine learning-derived risk scores need prospective validation in cohorts that were not used to build them. The third is combination design. Epidrugs and nanoformulated delivery systems will have to be tested alongside conventional chemotherapy, PARP inhibitors and immunotherapy rather than in isolation, since reversing epigenetic silencing is only useful when something else is available to exploit it. The fourth is restoration of microbial homeostasis through precision probiotics, postbiotics and periodontal treatment, which is the least developed of the four and rests on the weakest mechanistic footing.

## Data Availability

No new data were created or analyzed in this study. Data sharing is not applicable to this article.
